# Whole-genome epidemiology links phage-mediated acquisition of a virulence gene to the clonal expansion of a pandemic *Salmonella enterica* serovar Typhimurium clone

**DOI:** 10.1099/mgen.0.000456

**Published:** 2020-10-28

**Authors:** Eleonora Tassinari, Matt Bawn, Gaetan Thilliez, Oliver Charity, Luke Acton, Mark Kirkwood, Liljana Petrovska, Timothy Dallman, Catherine M. Burgess, Neil Hall, Geraldine Duffy, Robert A. Kingsley

**Affiliations:** ^1^​ Quadram Institute Bioscience, Norwich Research Park, Norwich, UK; ^2^​ Teagasc Food Research Centre, Ashtown, Dublin 15, Ireland; ^3^​ Earlham Institute, Norwich Research Park, Norwich, UK; ^4^​ Animal and Plant Health Agency, Addlestone, UK; ^5^​ Gastrointestinal Bacteria Reference Unit, National Infection Service, Public Health England, London, UK; ^6^​ University of East Anglia, Norwich, UK

**Keywords:** epidemic, evolution, phylogenomics, *Salmonella*, *sopE*

## Abstract

Epidemic and pandemic clones of bacterial pathogens with distinct characteristics continually emerge, replacing those previously dominant through mechanisms that remain poorly characterized. Here, whole-genome-sequencing-powered epidemiology linked horizontal transfer of a virulence gene, *sopE*, to the emergence and clonal expansion of a new epidemic *Salmonella enterica* serovar Typhimurium (*S*. Typhimurium) clone. The *sopE* gene is sporadically distributed within the genus *Salmonella* and rare in *S *. *enterica* Typhimurium lineages, but was acquired multiple times during clonal expansion of the currently dominant pandemic monophasic *S*. Typhimurium sequence type (ST) 34 clone. Ancestral state reconstruction and time-scaled phylogenetic analysis indicated that *sopE* was not present in the common ancestor of the epidemic clade, but later acquisition resulted in increased clonal expansion of *sopE*-containing clones that was temporally associated with emergence of the epidemic, consistent with increased fitness. The *sopE* gene was mainly associated with a temperate bacteriophage mTmV, but recombination with other bacteriophage and apparent horizontal gene transfer of the *sopE* gene cassette resulted in distribution among at least four mobile genetic elements within the monophasic *S *. *enterica* Typhimurium ST34 epidemic clade. The mTmV prophage lysogenic transfer to other *S. enterica* serovars *in vitro* was limited, but included the common pig-associated *S *. *enterica* Derby (*S*. Derby). This may explain mTmV in *S*. Derby co-circulating on farms with monophasic *S*. Typhimurium ST34, highlighting the potential for further transfer of the *sopE* virulence gene in nature. We conclude that whole-genome epidemiology pinpoints potential drivers of evolutionary and epidemiological dynamics during pathogen emergence, and identifies targets for subsequent research in epidemiology and bacterial pathogenesis.

## Data Summary

Whole-genome sequence data used for our analyses are freely available from the National Center for Biotechnology Information Sequence Read Archive (NCBI-SRA) under the BioProject numbers PRJNA605768 and PRJNA635911, and as indicated in Supplementary Material. In addition to those sequences previously reported, an additional 34 sequences of *Salmonella enterica* subsp. *enterica* serovars, isolated from pig farms from which *sopE*-positive monophasic *S*. Typhimurium sequence type (ST) 34 was also isolated, were determined in order to investigate the potential mTmV-mediated transmission of *sopE* (Table S1, available with the online version of this article). The previously reported whole-genome sequences of 1697 *S*. Typhimurium (and monophasic variant *S*. Typhimurium) from human clinical infections in England and Wales between 2012 and 2016 were used to investigate the population structure and distribution of *sopE* (Table S2). We determined the whole-genome sequence of an additional 49 monophasic *S*. Typhimurium ST34 strains isolated from UK pig farms between 2006 and 2015 in order to estimate a time-scaled phylogeny and ancestral state reconstruction of the *sopE* gene (Table S3). To investigate monophasic *S*. Typhimurium ST34 isolates from diverse sources (humans, food, environment, livestock, companion animals and wild animals), the whole-genome sequence of 442 isolates from 29 countries were investigated (Table S4). To place isolates tested for lysogenic conversion by mTmV in a phylogenetic context we constructed a maximum likelihood tree based on whole genome sequence of representative strains (Table S5).

**Impact Statement**

An understanding of the evolutionary processes associated with the emergence of new bacterial pathogens is important for the development of rational intervention policies aimed at limiting their spread and impact on human health. We previously reported the sporadic distribution of the *sopE* virulence gene in a recently emerged pandemic clone of monophasic *S.* Typhimurium sequence type (ST) 34. We now report that *sopE* was acquired on multiple occasions after initial clonal expansion of the epidemic clade, mediated by mTmV phage. Strains carrying the *sopE* virulence gene occurred in closely related clusters, consistent with increased clonal expansion, perhaps due to enhanced fitness. The mTmV phage was capable of transferring widely within diverse *S*. Typhimurium genotypes *in vitro* but, to date, little evidence has been found of this having occurred frequently outside of the monophasic *S*. Typhimurium ST34 epidemic clade in nature. Transfer to strains of diverse *S. enterica* serovars was limited *in vitro*, but *S*. Derby strains isolated on pig farms at which monophasic *S*. Typhimurium ST34 containing mTmV were present also contained this mTmV in their genome, suggesting transfer does occur in nature. This is believed to be the first report of frequent acquisition of a virulence gene stimulating clonal expansion of a new bacterial pathogen clone.

## Introduction

Non-typhoidal *Salmonella* is a major cause of foodborne gastroenteritis worldwide associated with a considerable disease burden and economic losses [1]. *S.* Typhimurium is one of the most common serovars responsible for human and animal infections [2, 3]. In Europe, the epidemiology of *S*. Typhimurium over the past 60 years has been characterized by successive waves of dominant multi-drug resistant (MDR) clones that were replaced every 10–15 years [4, 5]. Since around the year 1965, DT9, DT204, DT193, DT104 and, currently, monophasic *S*. Typhimurium (*S*. 4,[5],12;i:-) of sequence type (ST) 34 were sequentially the most common *S*. Typhimurium types. These epidemic clones share a similar antimicrobial-resistance profile, generally including resistance to ampicillin, chloramphenicol, sulphonamides, streptomycin and tetracycline, and in some cases additional resistance.

Monophasic *S*. Typhimurium ST34 emerged in Europe around the year 2005, initially in pig populations, and later in cattle and to a lesser extent in poultry [2, 6–9], replacing the previously dominant DT104 clone [10, 11]. The epidemic spread in pigs was mirrored by its rapid emergence as the dominant *S*. Typhimurium clone isolated from human clinical infections, highlighting the importance of pigs as a zoonotic reservoir [12]. Monophasic *S*. Typhimurium ST34 typically contain a chromosomally located composite transposon that encodes genes conferring resistance to ampicillin, streptomycin, sulphonamide and tetracycline (ASSuT) [13]. However, additional resistance to clinically relevant antimicrobials, following the acquisition of plasmids, has also been reported [14–16]. Monophasic *S*. Typhimurium ST34 has now been reported worldwide [9, 17].

Although multi-drug resistance appears to be a key characteristic for the epidemic spread of dominant clones, this characteristic does not differentiate successive clones; therefore, additional drivers for the emergence of a new clone remain to be identified. Little is known about what drives the replacement of the epidemic MDR clones of *S*. Typhimurium, but it is clear that horizontal gene transfer (HGT) is key to the evolution by introducing new genes or clusters of genes that have the potential to significantly modify the characteristics of the pathogen [18]. Monophasic *S*. Typhimurium ST34 acquired enhanced resistance to copper through a novel integrative conjugative element termed *Salmonella* genomic island 4 (SGI-4) [9, 19, 20]. Resistance to copper may have contributed to the epidemic spread of monophasic *S*. Typhimurium ST34, since this transition metal is widely used at therapeutic levels in the livestock industry as a growth-promoter, due to its antimicrobial activity [9, 19, 21].

Temperate bacteriophages represent an important source of HGT that can modify the characteristics of bacteria through cargo genes [22], and account for a greater proportion of the lineage-specific accessory genome in *S*. Typhimurium than either plasmids or non-phage chromosomal genes [23]. Monophasic *S*. Typhimurium ST34 acquired the *sopE* virulence gene through HGT mediated by lysogenic conversion of the novel temperate bacteriophage mTmV [9]. A variant of mTmV, designated mTmV2, sharing approximately the 50 % of the genome with mTmV was later described in monophasic *S*. Typhimurium ST34 isolates from Italy [24].

The *sopE* gene encodes a guanine nucleotide exchange factor (GEF) that activates the Rho-GTPases Cdc42 and Rac-1 in the host cells, thereby inducing actin-mediated membrane ruffling, which promotes microbial uptake [25]. The activity of SopE is also detected by the inflammasome resulting in the activation of the NFκB-dependent inflammatory process [26]. Therefore, SopE contributes to inflammation that boosts *S*. Typhimurium growth in the gut lumen [27]. Additionally, SopE has been found to be involved in the early stages of intracellular replication inside *Salmonella *-containing vacuoles in macrophages [28]. The *sopE* gene has been reported in multiple *S.* serotypes, but is rarely present in *S*. Typhimurium. A notable exception is its presence in the MDR *S*. Typhimurium DT204 epidemic in Europe on a P2-like prophage SopEΦ [5].

To investigate the potential role of the *sopE* gene in the epidemic spread of monophasic *S*. Typhimurium ST34, we applied a whole-genome-sequence-based epidemiological approach to establish the ancestral history of its horizontal and vertical transfer, and its impact on population structure. Furthermore, we investigated the mobile genetic elements (MGEs) associated with *sopE* and their potential to disseminate this gene within serotypes of *S. enterica *.

## Methods

### Bacterial strains and growth conditions

For routine culture, strains were grown in 5 or 10 ml of LB broth (Miller) at 37 °C with shaking (200 r.p.m.) for 16–17 h. Monophasic *S*. Typhimurium ST34 strain 0343A was isolated from a pig farm in 2012 [16]. Allelic exchange mutants of *S*. Typhimurium ST34 strain 0343A in which the *aph* (strain ET280) or *cat* gene (strain ET141) were inserted between the 3′ end of *sopE* and the adjacent gene were generated by recombineering, based on that previously described [29, 30]. The *cat* or *aph* genes were amplified using oligonucleotide primers annealing to pKD4 and pKD3 plasmids, respectively, with 50 nucleotides homologous to the flanking sequence of the insertion site (Table S6). Mutant strains were routinely cultured in LB broth (Miller) supplemented with either kanamycin (50 µg ml^−1^) or chloramphenicol (25 µg ml^−1^). Monophasic *S*. Typhimurium ST34 strain S01569-10 isolated in the UK in 2010 [9] was tested for the presence of multiple copies of *sopE* by PCR using primers that annealed outside the *sopE* cassette conserved between the distinct bacteriophages (Table S6). A collection of representative *S*. Typhimurium and *S.* serovar isolates (Tables 1 and S1) were tested for susceptibility to mTmV phage lysogeny (see below).

### Lysogenic transfer experiments

Phage transfer experiments were performed to assess the ability of mTmV to establish lysogeny in a collection of *S. enterica* isolates. *S*. Typhimurium ST34 strain ET280 or ET141 carrying the mTmV prophage in which the *sopE* gene had been replaced by the *aph* or *cat* gene conferring antibiotic resistance was grown in 5 ml LB broth at 37 °C for 16–17 h with shaking (200 r.p.m.). Cells were pelleted by centrifugation (10 min at 4000 r.p.m.), and the supernatant collected and filter sterilized using a 0.22 µm pore size membrane. The sterility of the filtered supernatant was checked by culture of 0.1 ml on LB agar plates for 16 h at 37 °C. The recipient strains were cultured at 37 °C for 16–17 h with shaking (200 r.p.m.), adjusted to an OD_600_ of 0.01 in Falcon tubes with 4 ml sterile LB broth and 1 ml sterile supernatant and incubated at 37 °C for 24 h with shaking (100 r.p.m.). Serial dilutions were cultured on LB agar plates with and without chloramphenicol or kanamycin as appropriate and incubated for 16 h at 37 °C. The frequency of lysogeny is expressed as the number of lysogen c.f.u. as a proportion of recipient c.f.u. Lysogenic isolates were checked for the integration of mTmV prophage in the *thrW* locus by PCR using oligonucleotide primers annealing to the flanking sequences of the left and right mTmV attachment sites (Table S6).

### Preparation of genomic DNA and sequencing

Genomic DNA for short-read sequencing was extracted using a Wizard genomic DNA purification kit (Promega). The DNA for long-read sequencing was extracted by alkaline lysis and purification was performed using phenol-chloroform, as previously described [9]. Libraries were prepared with the DNA from 34 *S *. *enterica* subsp. *enterica* strains (Tables 2 and S1) using a Nextera XT DNA library preparation kit (Illumina) and sequenced on a NextSeq 500 system (Illumina), according to the manufacturer's instructions. LITE libraries with the DNA of 49 monophasic *S*.Typhimurium ST34 (Table S3) were generated and sequenced at the Earlham Institute using a HiSeq 2500 system (Illumina), as described previously [31]. Long-read whole-genome sequencing of strain S01569-10 [9] was performed using a PacBio RS system (Pacific Biosciences) at the Earlham Institute and long-read sequence assembled, as previously described [23].

**Table 1.** *S*. Typhimurium isolates tested for lysogenic conversion by mTmV phage The strain name, phage type, ST, host range and epidemiology, and accession number are reported. nd, Not determined.

Strain name	Phage type	ST	Host range and epidemiology	Accession no.
SL1344	DT44	ST19	Broad range, MDR clone epidemic in 1970–1985	FQ312003.1
NCTC 13348	DT104	ST19	Broad range, MDR clone epidemic in 1990–2010	HF937208.1
D23580	nd	ST313	Human-adapted, epidemic disseminated disease in Africa	FN424405.1
01960-05	U288	ST19	Porcine, MDR clone epidemic in pigs	ERR029231
S07292-07	U308	nd	Porcine	ERR029234
11020-1996	DT193	nd	Porcine	ERR028308
L01157-10	DT8	ST19	Duck/goose-associated	PRJEB34595
033715	DT2	ST98	Wild birds (pigeon), endemic in pigeon	ERR028071
2882-06	DT99	nd	Wild bird (pigeon), endemic in pigeon	ERR024407
SO7676-03	DT56	ST568	Wild birds (passerines)	ERR029222
4179-2001	DT121	nd	Wild birds (passerines)	ERR028301

**Table 2.** Number of isolates and farms of isolation of each of the *S. enterica* serovars sequenced

Serovar	No. of isolates	Farm	*sopE* (*n*)	mTmV (*n*)
Anatum	1	A	–	–
Derby	15	B, C, D, E, G, I, J	4	4
Dublin	3	H, I	2	–
Infantis	2	E	–	–
London	6	I, J	–	–
Stanley	1	B	–	–
Tennessee	3	J	–	–
Typhimurium Copenhagen	3	G, I	–	–

### Phylogenetic reconstruction and sequence analyses

Maximum-likelihood (ML) methods were used to infer the phylogenetic relationships of microbial isolates and single genes. Whole-genome-based phylogenies were built on recombination-purged SNPs in the core genome, with reference to *S*. Typhimurium SL1344, an ST19 strain from outside of the monophasic *S*. Typhimurium ST34 epidemic clade [32]. For the phylogenetic trees in Figs 2 and 3, the genome of *S*. Typhimurium LT2 [33] was included as an outgroup to define the root of the monophasic *S*. Typhimurium ST34 clade. The SNPs were identified with Snippy software v3.0 [34], which aligned the raw reads to a reference sequence using bwa-mem [35], and variant calling and SNP filtering with Freebayes [36] and vcflib/vcftools [37]. Recombinant regions were excluded using Gubbins v2.3. [38]. The ML tree was then computed with RAxML v8.0.20 using a general time reversible (GTR) substitution model with gamma correction for among-site rate variation with rapid bootstrapping [39]. Patristic distances were computed using the cophenetic function in the ape R package [40].

Phylogroups of biphasic *S*. Typhimurium from UK clinical samples were identified with the R package rPinecone [41], which defines sub-lineages within a phylogenetic tree using a root-to-tip distance approach. We used a distance threshold of 150 SNPs to define clusters. ML phylogeny of the *sopE* gene was computed with RAxML-NG v0.9.0 using the DNA substitution model HKY+FO+G [42].

The diversity of *sopE* sequences included in the analysis has been described elsewhere [43]: *S. enterica* subspecies IV strains SARC9 (accession number AF378111) and SARC10 (accession number AF378112), *S. enterica* subspecies VII strains SARC15 (AF378113) and SARC16 (accession number AF378112), *S. enterica* subspecies I serovar Hadar strain X3230 (accession number AY034828), Gallinarum strain X3796 (AF380340), Dublin (L78932), Typhi strains SARC2 (accession number AF378115) and X3744 (accession number AF153829.1), Typhimurium strain SL1344 (accession number AF043239.1), monophasic *S*. Typhimurium strain S04698-09 (accession number NZ_LN999997.1). Phylogenetic tree visualization, manipulation and annotation were performed using ggtree [44], iTOl [45] and FigTree v1.4.2 [46]. Nucleotide sequences of interest were detected in raw short-read sequence data using ariba v2.8.1 [47] or srst2 v0.2.0 software [48] with customized databases. Matches with >90 % sequence coverage and <10 % of sequence divergence were reported as present. Alignments of whole-genome-sequence assemblies were performed with the blast algorithm [49] and visualized by plotting the comparison using genoPlotR [50]. Gene copy number was estimated from high-depth (36–500×), short-read whole-genome sequences by calculating the ratio between the number of reads mapping to *sopE* and the number of reads mapping to *sopE2*, which shares 98 % of nucleotide sequence (70 % of identity) with *sopE*.

### Time-scaled phylogeny and ancestral state reconstruction

A dated phylogenetic tree was inferred using BactDating [51]. Before inferring the dates of the ancestral nodes, the presence of a molecular clock signal within the collection of isolates was tested by evaluating the correlation of the root-to-tip distance with the isolation dates by regression analysis. In total, 10^6^ Markov chain Monte Carlo iterations were performed to ensure convergence. The effective sample size of the α, μ and σ parameters was >200. Ancestral state reconstruction estimated the state for a character at ancestral nodes of a phylogenetic tree. To this end, stochastic character mapping was performed with the R package phytools [52] using the empirical Bayes method, in which the prior distribution was estimated from the data, using an equal rate model. Five hundred simulations were performed, the simulated mapped states were then merged and the character states with the relative posterior probability were plotted on the phylogenetic tree.

### Statistical analyses

A Mann–Whitney U test using GraphPad Prism v5.04 was performed to test the null hypothesis that the distribution of the patristic distances between two groups of isolates is identical. Permutation tests (5000 simulations) were performed in R [53] to test the null hypothesis that the difference in mean patristic distance between groups of isolates identified based on gene presence/absence did not depend on the distribution of the gene across the phylogeny. The *P* value was calculated as how many times the simulated difference exceeded the observed one. Fisher’s exact test (GraphPad Prism v5.04) was performed to test the presence of non-random association between the presence of *sopE* and the proportion of isolates in transmission clusters.

## Results

### *sopE* gene is rare in *S*. Typhimurium lineages

**Fig. 1.**
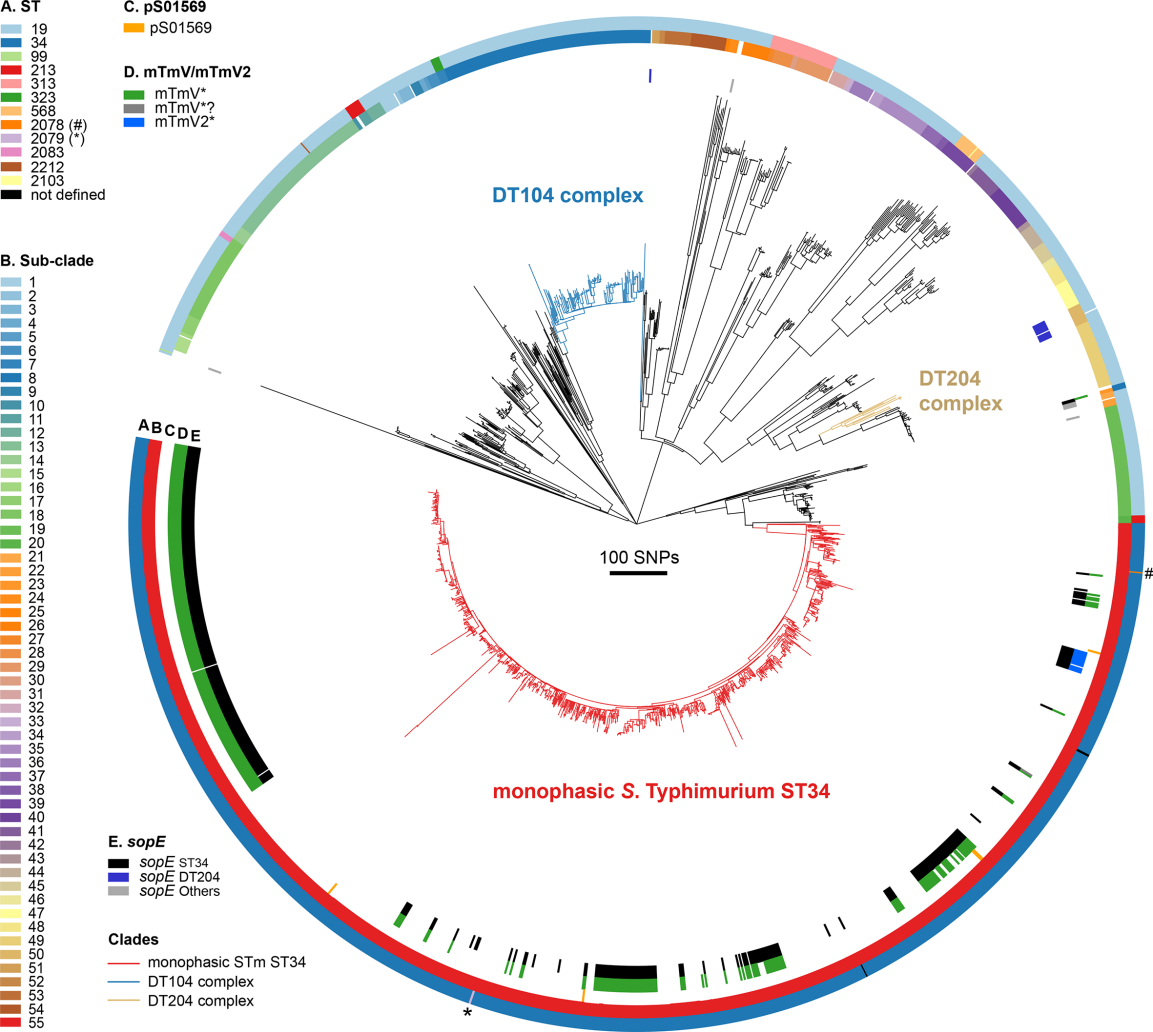
Population structure of *S*. Typhimurium from human clinical cases of infection in England and Wales and distribution of *sopE*. ML phylogenetic tree of *S*. Typhimurium and monophasic *S*. Typhimurium isolated from UK human salmonellosis subjects between 2012 and 2016, showing the distribution of the *sopE* gene, of mTmV/mTmV2 and pS01569 phages, and the population structure based on a root-to-tip SNP-threshold clustering method and ST. The tree is of 1697 *S *. *enterica* Typhimurium and monophasic *S*. Typhimurium isolates from UK clinical infections and was generated using core-genome SNPs identified with reference to *S*. Typhimurium SL1344. The circles represent (A) the ST, (B) epidemiologically relevant phylogroups using a root-to-tip distance threshold of 150 SNPs, (C) the distribution of the prophage plasmid pS01569, (D) mTmV and related sequences, with poor coverage and/or polymorphisms indicated as mTmV*?, and (E) the distribution of six *sopE* allelic variants reported as three variables – monophasic *S*. Typhimurium ST34 allele, *S*. Typhimurium DT204 complex allele, or one of the four alternative alleles. Four strains within the monophasic *S*. Typhimurium ST34 lineage were not ST34: two were predicted to be ST2078 and ST2079, which differ by a single-locus SNP from ST34, while the remaining two were of undefined ST.

The *sopE* gene was previously reported in isolates of DT44, DT49 and DT204, together referred to as the DT204 complex and responsible for an epidemic in livestock from around 1974 to 1990 in Europe [5, 54]. To investigate the distribution of *sopE* in 1697 *S*. Typhimurium (and monophasic variant *S*. Typhimurium) from human clinical infections in England and Wales between 2012 and 2016 [19], we reconstructed a ML phylogenetic tree based on recombination-purged sequence variation in the core genome (Fig. 1, Table S2). The population structure was characterized by multiple lineages extending from a common ancestor in a star topology. A total of 74 epidemiologically relevant phylogroups were identified based on the divergence in the recombination purged core genome, similar to that observed for the *S*. Typhimurium DT104 and *S*. Typhimurium DT204 epidemic clades (<150 SNPs). Phylogroups included 55 clades and 19 lineages with a single representative genome (Fig. 1). The *sopE* gene was present in 7 of the 74 phylogroups, outside of the monophasic *S*. Typhimurium ST34 clade. Of 21 non-ST34 isolates with *sopE*, 11 were in the DT204 complex, consistent with being the vestiges of the previous epidemic. Two closely related *S*. Typhimurium isolates present in a single phylogroup also harboured *sopE* and were ST34, despite being present on a lineage distinct from the monophasic *S*. Typhimurium ST34 clade. The ST34 genotype outside the monophasic *S*. Typhimurium ST34 phylogroup may be the result of a recombination event.

### Multiple acquisition of *sopE* by monophasic *S*. Typhimurium ST34 temporally coincided with clonal expansion

We previously reported the sporadic distribution of the *sopE* gene in monophasic *S*. Typhimurium ST34 isolates from the UK and Italy [9]. However, it was not known whether *sopE* was acquired multiple times by monophasic *S*. Typhimurium ST34 or, alternatively, acquired once and subsequently deleted on multiple occasions, during clonal expansion. To investigate the evolutionary history of *sopE* in the monophasic *S*. Typhimurium clade, we inferred the ancestral state of the most recent common ancestor (MRCA) and hypothetical intermediary descendants, with respect to the presence of *sopE*, for 62 monophasic *S*. Typhimurium ST34 strains isolated between 2006 and 2015 from UK pig farms (Table S3). We focussed on UK isolates from pigs, because this host is reported to be the primary population associated with the epidemic spread of monophasic *S*. Typhimurium ST34, and we limited our analysis to the UK to capture as much as possible spread in a single population.

**Fig. 2.**
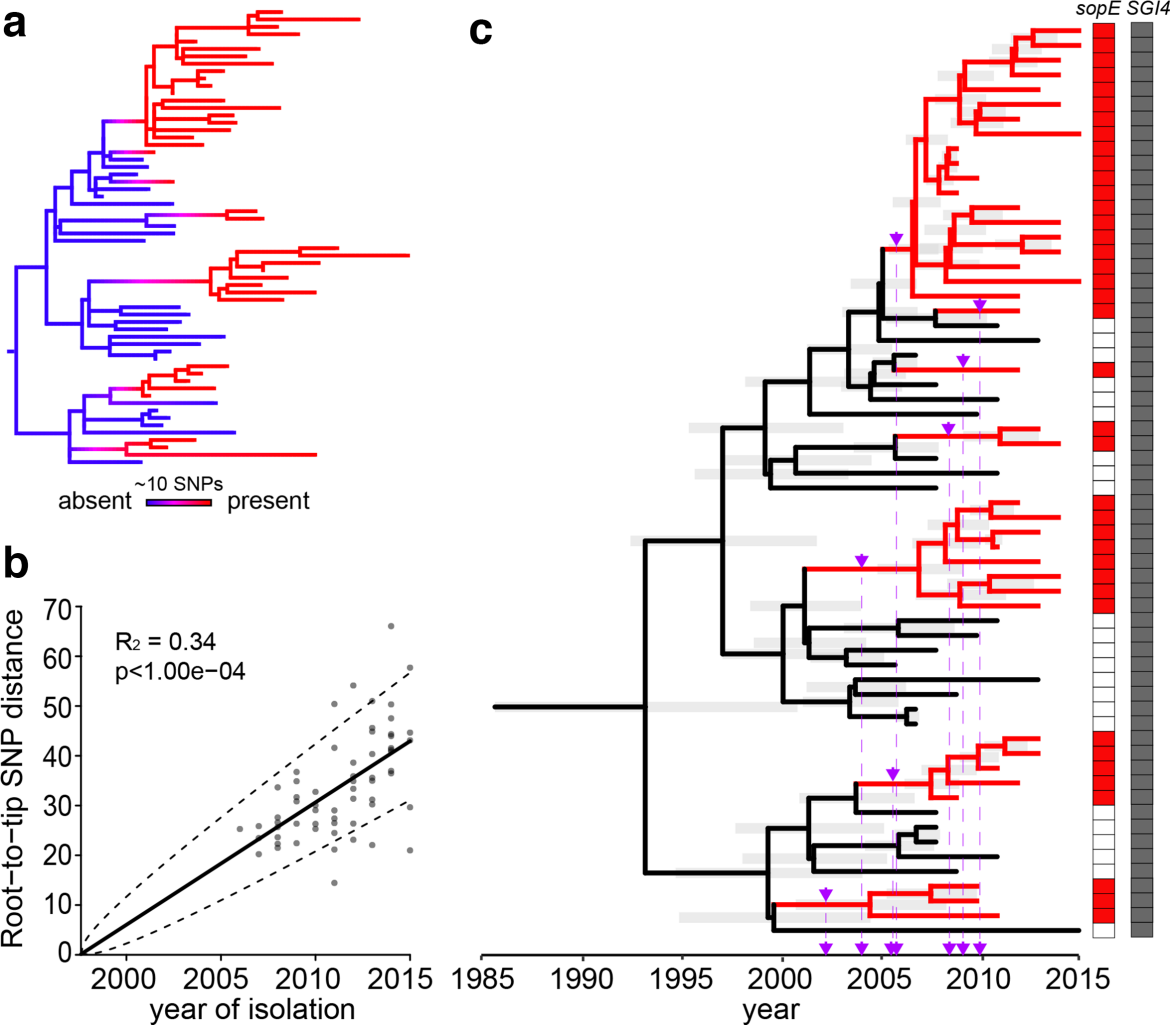
Ancestral state reconstruction of *sopE* with time-scaled phylogeny. Variation in the whole-genome sequences of *S*. Typhimurium isolated from UK pigs between 2006 and 2015 was used for ancestral state reconstruction and time-scaled phylogenetic trees. (a) ML phylogenetic tree based on recombination-purged variation in the core-genome sequence showing the reconstruction of the ancestral character state of *sopE*. The colour gradient displays the probability associated with *sopE* presence/absence inferred from 500 simulations. The number of SNPs (~10) is indicated by the length of the bar, and was estimated from the genetic distance as a proportion of SNP sites used to reconstruct the tree. (b) Root-to-tip regression analysis assessing the presence of a significant temporal signal within the isolate collection. The plot shows the correlation between the number of SNPs accumulated and the year of isolation, which was statistically significant (*P* value 0.0001). The coefficient of determination (r^2^), which indicates how well the model fits the data and reflects the among-lineage rate variation, was 0.34. (c) Dated phylogenetic tree inferred from the ML phylogenetic tree in (a) showing the events of *sopE* acquisition (purple arrows).

A ML phylogenetic tree based on 1095 core-genome recombination-purged SNPs with reference to *S*. Typhimurium strain SL1344 was reconstructed. The *sopE* gene, all with identical nucleotide sequence, was detected in short-read sequence of 39 isolates in discrete clades in the phylogenetic tree. Ancestral state reconstruction was used to infer the character change (*sopE* acquisition and loss) along the phylogeny by stochastic character mapping (Fig. 2a). The MRCA and descendants at deeply rooted nodes of the tree were predicted to be negative for *sopE*, consistent with at least seven gene acquisition events during the clonal expansion of the *S*. Typhimurium ST34 epidemic clade.

Accumulation of SNPs through the rooted phylogeny had a statistically significant temporal signal; therefore, we were able to infer the approximate time of acquisition of *sopE*. Linear regression analysis of the root-to-tip accumulation of recombination-purged SNPs indicated a fraction of variance explained by the model, R^2^=0.34, with a significant *P* value (*P*<0.0001), based on a permutation test (Fig. 2b). This corresponded to a mutation rate of 6.8×10^−7^ substitutions per site per year. Bayesian inference was used to estimate the ancestral dates of nodes. The MRCA of isolates from UK pigs in this collection was around 1994 (Fig. 2b, c). The earliest acquisition of the *sopE* gene was around the year 2002, and the most recent around 2010. This was consistent with the view that a *sopE*-negative monophasic *S*. Typhimurium ST34 was present for several years before acquiring the *sopE* gene by multiple independent HGT events that temporally coincided with the first reports of the epidemic clone from UK pig farms around 2006, and subsequent rapid spread [9].

**Fig. 3.**
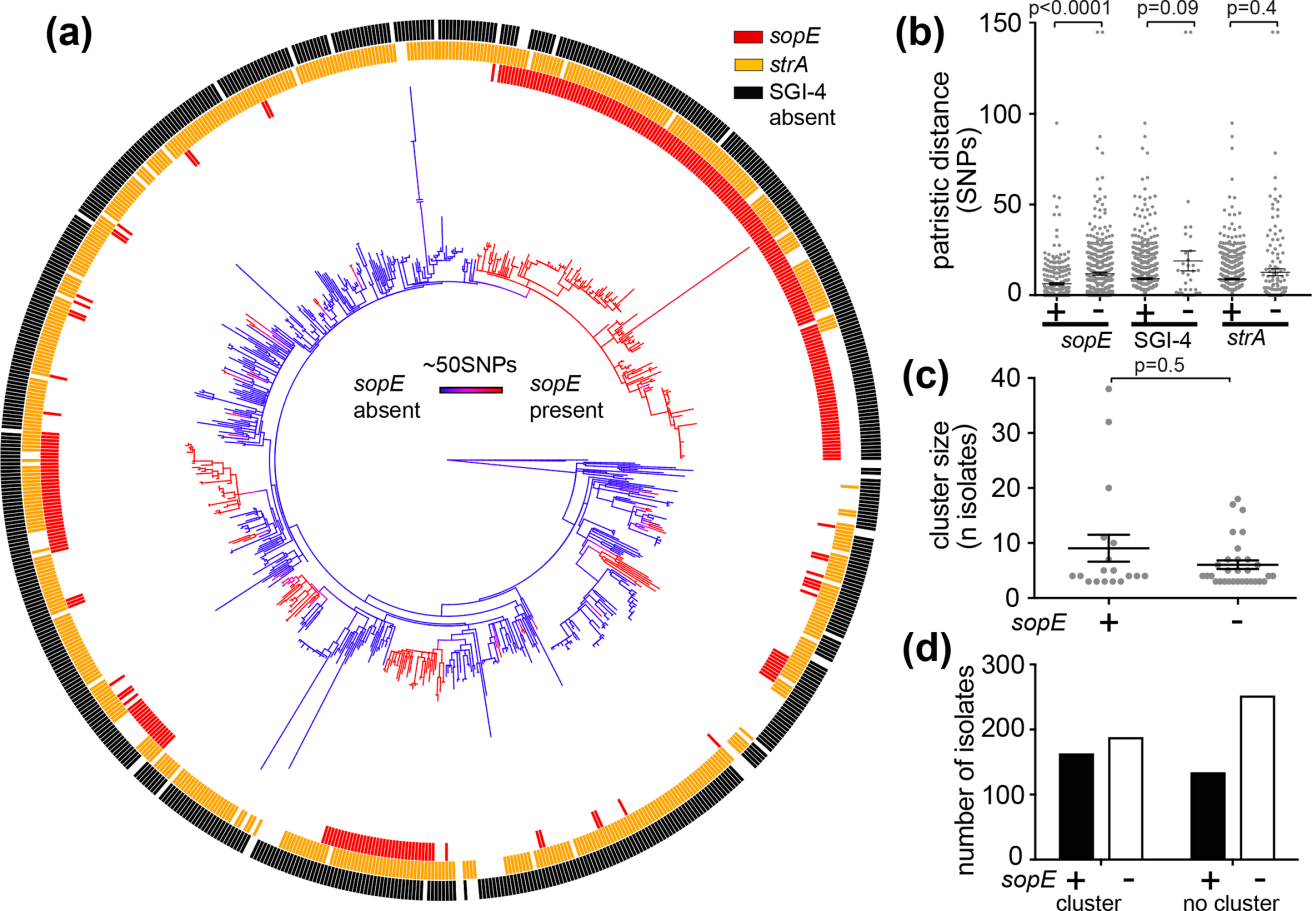
Multiple acquisitions of *sopE* by monophasic *S*. Typhimurium from UK human salmonellosis subjects were associated with clonality and transmissibility of the isolates. (a) ML phylogenetic tree of monophasic *S*. Typhimurium ST34 isolated in the UK from human infections between April 2014 and March 2015 displaying ancestral state reconstruction for *sopE* and the distribution of *sopE* and *strA* genes and of the SGI-4. The posterior probability associated with *sopE* presence or absence inferred by stochastic character mapping is plotted on the tree as a colour gradient of blue (absent) to red (present). (b) Distribution of pairwise patristic distance, expressed as the number of SNPs, for isolates encoding and lacking *sopE*, the SGI-4 and *strA*. Isolates harbouring *sopE* displayed significantly lower patristic distances than isolates without *sopE* (Mann–Whitney test, *P* value 0.0001), while the difference of patristic distance between isolates positive and and negative for the SGI-4 and strA was not statistically significant (Mann–Whitney test, SGI-4 *P* value=0.09, *strA P* value=0.4). (c) Size of transmission clusters of *sopE*-positive and *sopE*-negative monophasic *S*. Typhimurium isolates. Clusters were defined as groups of ≥3 isolates sharing the same SNP address at a five single-linkage SNPs level. Isolates with *sopE* were associated with larger transmission clusters, but the difference was not statistically significant (Mann–Whitney test, *P* value 0.5). (d) Number of *sopE*-positive and *sopE*-negative isolates that were or were not associated with transmission clusters. Isolates with *sopE* were significantly more associated with transmission clusters (Fisher’s exact test, *P* value 0.01).

To investigate the distribution of *sopE* in monophasic *S*. Typhimurium ST34 from human clinical infections in the UK, we analysed the whole-genome sequence of a collection of 737 isolates from a 12 month period (April 2014 – March 2015) (Fig. 3, Table S2). A ML phylogenetic tree based on 4423 recombination-purged core-genome SNPs, with reference to *S*. Typhimurium SL1344, was reconstructed to investigate the distribution of *sopE* within the phylogenetic context of the isolates. The *sopE* gene was present in the genome of 297 isolates (41%) in 15 discrete clades, and a further 15 singleton leaves on the tree (red lineages in Fig. 3a). Ancestral state reconstruction using probabilistic models indicated that *sopE* was acquired in 33 independent events, and lost in 7 events, in this dataset (Fig. 3a). Similar to the topology of the monophasic *S*. Typhimurium ST34 from pigs, most isolates containing *sopE* (95%) were present in low diversity clusters, consistent with clonal expansion. In contrast, loss of *sopE* occurred sporadically with little evidence of subsequent clonal expansion.

### Acquisition of *sopE* was followed by clonal expansion consistent with positive selection

The high frequency of *sopE* in clinical isolates, resulting from 33 independent acquisition events, suggested that *sopE* may be beneficial for monophasic *S*. Typhimurium ST34 circulation in livestock or in human clinical infection (Fig. 3). We considered that if the acquisition of *sopE* conferred a fitness advantage to transmission between hosts, then this would result in the clonal expansion *sopE*-positive clones as a result of positive selection. Therefore, we tested the hypothesis that *sopE* enhanced the fitness of the isolates by comparing the degree of clonality of *sopE*-positive and *sopE*-negative isolates. We did this by determining the mean patristic distance (genetic distance to its closest relative) as an estimate of clonality in 737 human clinical isolates of monophasic *S*. Typhimurium ST34. Isolates with *sopE* exhibited significantly lower patristic distance (6.2 SNPs) compared to *sopE*-negative isolates (11.8 SNPs) (Mann–Whitney test, *P* value<0.0001), suggesting that acquisition of *sopE* resulted in clonal expansion, consistent with positive selection (Fig. 3b).

We next investigated the effect on the population structure of loss of SGI-4 or *strA*. In contrast to *sopE*, the distribution of SGI-4 and *strA* was consistent with their loss from the genome during clonal expansion and, therefore, presented the opportunity to test whether differences in the patristic distance were due to recent genotypic variation, rather than selection (Fig. 3a). SGI-4 is an integrative conjugative element encoding heavy metal resistance loci and may be important to the spread of monophasic *S*. Typhimurium ST34 in pig populations, where copper supplementation of feed at therapeutic levels is common. Only 37 of 737 monophasic *S*. Typhimurium ST34 strains of the UK clinical isolates lacked the genomic island following 25 apparent deletion events. Isolates in which SGI-4 had been deleted had a higher mean patristic distance than isolates lacking the sequence, 19 SNPs and 9.1 SNPs, respectively (Fig. 3b, Mann–Whitney test, *P* value=0.09), consistent with a loss of fitness resulting from the loss of SGI-4. The *strA* gene confers resistance to streptomycin and had a sporadic distribution within the monophasic *S*. Typhimurium ST34 clade. It was present flanked by IS*26* elements on a composite transposon that encoded a number of AMR genes and is thought to have been acquired by the MRCA of monophasic *S*. Typhimurium ST34 [13]. The mean patristic distance of isolates that had lost the *strA* gene was not statistically significant (Fig. 3b; Mann–Whitney test, *P* value=0.4), consistent with a lack of selection for the loss of the *strA* gene.

In order to test whether the observed difference in mean patristic distance for isolates either having or lacking *sopE* was observed by chance, we randomly reassigned the *sopE,* SGI-4 and *strA* genotype to genomes at the original frequency. The simulated difference of the mean patristic distance for each genotypic variant was significantly different from that observed (*sopE P* value 0; SGI-4 *P* value 0.005; *strA P* value 0.005), indicating that the observed changes in patristic distance were not due to chance.

The rate of transmission has previously been reported to be directly proportional to the genetic similarity of the isolate; therefore, the proportion of isolates that fall in transmission clusters can be used to estimate the transmissibility of a genotype [55, 56]. A molecular clustering method employing a SNP address has been used to define putative transmission clusters in epidemiological surveillance, as they are consistent with epidemiologically related isolates that share the same source [57]. We compared the number of *sopE*-positive and *sopE*-negative isolates potentially part of a transmission cluster. Candidate transmission clusters were defined as groups with three or more isolates sharing the same SNP address, with a 5 SNP distance threshold [58]. In our dataset of UK clinical isolates, 49 candidate transmission clusters were identified, of which 31 were *sopE*-negative monophasic *S*. Typhimurium ST34 and the remaining 18 *sopE*-positive monophasic *S*. Typhimurium ST34, reflecting the relative proportion in the population and the recent acquisition of *sopE*. The mean size of *sopE*-positive transmission clusters was greater than of *sopE*-negative clusters, although this did not reach statistical significance (Fig. 3c; Mann–Whitney U test, *P* value 0.5). However, fewer *sopE*-negative isolates were in potential transmission clusters than isolates present outside clusters on relatively extended branches in the phylogenetic tree. In contrast, more *sopE*-positive isolates were in potential transmission clusters than isolates present outside clusters (Fig. 3d). Indeed, the proportion of *sopE*-positive isolates that were in potential transmission clusters (<5 SNP clusters) was significantly greater than that of isolates lacking *sopE* (54.8 and 42.7 %, respectively; Fisher’s exact test, *P* value 0.001), suggesting that clones of monophasic *S*. Typhimurium ST34 with *sopE* had enhanced transmission fitness.

### Monophasic *S *. *enterica* Typhimurium ST34 acquired a novel *sopE* allelic variant

**Fig. 4.**
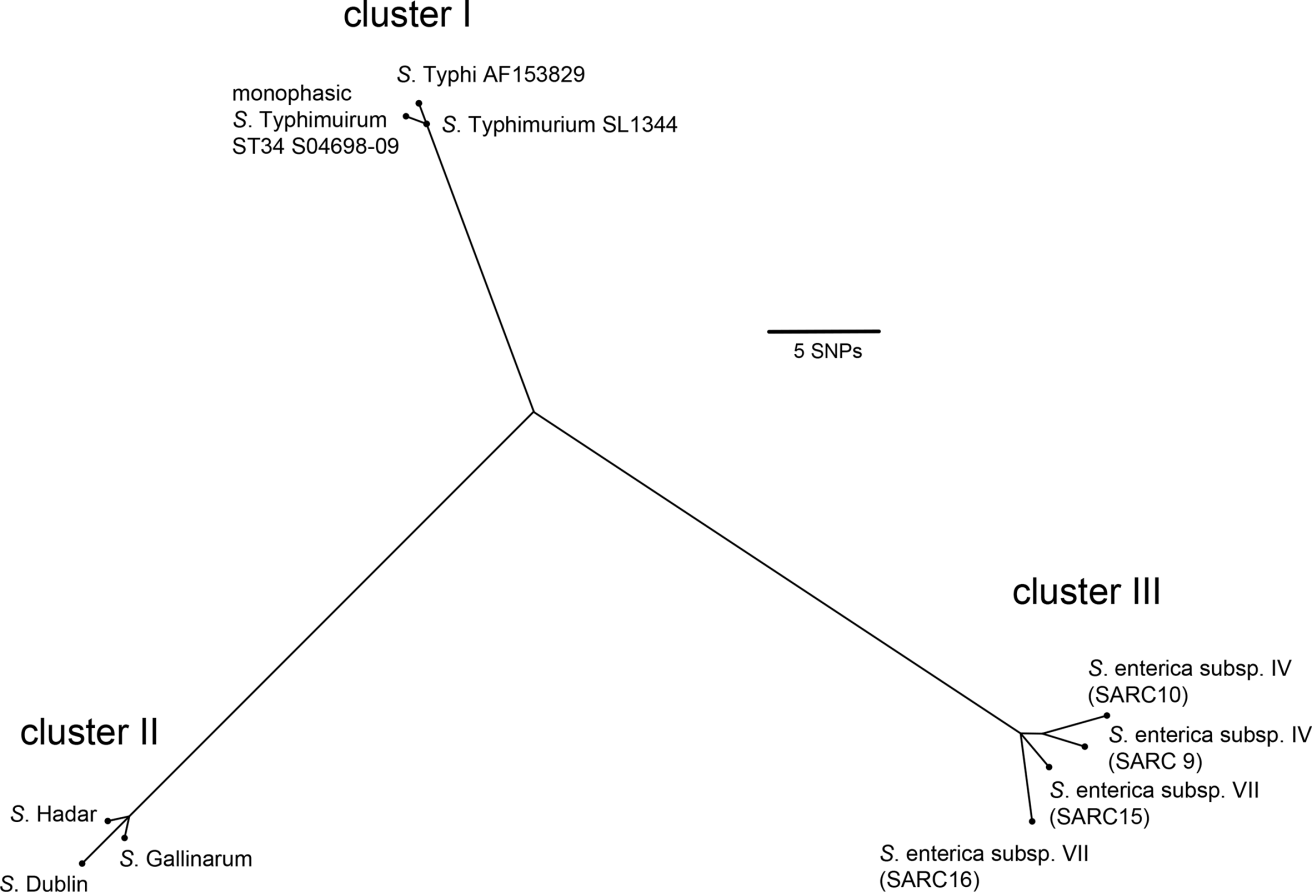
Phylogenetic relatedness of *sopE* from diverse *S. enterica* subspecies and *S. enterica* subsp. *enterica* serovars. The unrooted ML tree, generated with RAxML based on nucleotide variation, identified three clusters, with cluster I comprising the *sopE* sequence of monophasic *S*. Typhimurium ST34 from isolate S04698-09.

The *sopE* gene has a sporadic distribution within *S. enterica *, and is present as one of three alternative clusters of related alleles, termed I, II and III [43]. Cluster I alleles were present in *S*. Typhimurium strain SL1344 on the SopE phage and *S *. *enterica* Typhi, cluster II in *S *. *enterica* Gallinarum, *S *. *enterica* Dublin and *S *. *enterica* Hadar, and cluster III in *S. enterica* subspecies IV and VII. To investigate the phylogenetic relationship of the *sopE* gene from *S*. Typhimurium ST34 isolates with *sopE* from diverse *S. enterica* serotypes, reported previously [43], a ML phylogenetic tree was reconstructed based on variation in nucleotide sequence. The *sopE* allelic variant in monophasic *S *. *enterica* Typhimurium strain S04698-09 [9] was present in cluster I and differed from *S*. Typhimurium strain SL1344 *sopE* by one synonymous SNP at position 27 (Fig. 4). Comparison of the monophasic *S*. Typhimurium *sopE* with sequences in available databases indicated that it was identical to *sopE* from *S. enterica* serovar Miami strain ATCC BAA-1586 (accession number CP023468.1) [59]. Furthermore, sequences flanking *sopE* in monophasic *S*. Typhimurium ST34 and *S *. *enterica* Miami ATCC BAA-1686 were identical, but distinct to that flanking *sopE* in strain SL1344 (data not shown), suggesting they share the same common ancestor.

### *sopE* gene is associated with multiple MGEs in the monophasic *S *. *enterica* Typhimurium ST34 epidemic clade but predominantly prophage mTmV

The acquisition of *sopE* in monophasic *S*. Typhimurium ST34 has been reported to be associated with two related prophages mTmV and mTmV2 [9, 24]. Therefore, we investigated the association of each of these sequences with the *sopE* gene in monophasic *S*. Typhimurium ST34. The *sopE* gene was most frequently associated with mTmV in the UK, and globally. A total of 90 % (267/297) of *sopE*-positive monophasic *S*. Typhimurium ST34 isolated from clinical infections in the UK between April 2014 and March 2015 contained the mTmV prophage. In contrast, 2.7 % (8/297) had the mTmV2 prophage, and 7.4 % (22/297) had neither mTmV nor mTmV2 (Fig. 1, Table S2). Of 442 monophasic *S*. Typhimurium ST34 isolates from diverse sources (human, food, environment, livestock, companion animals and wild animals) in 29 countries, 25 % (*n*=110) encoded the *sopE* gene (Table S4). In this global collection of isolates, the *sopE* gene was associated with mTmV in 63.6 % (*n*=70) of the *sopE*-positive monophasic *S*. Typhimurium ST34 isolates that were isolated from 13 different countries across Europe, Africa, Asia and America. In contrast, mTmV2 was detected in 20.9 % (*n*=23) of isolates encoding *sopE*, the majority of which were from Italy. The remaining 18.2 % (*n*=20) of isolates from seven different countries in Europe, Asia or America were not associated with mTmV or mTmV2.

### mTmV prophage is rare in *S *. *enterica* outside of the monophasic *S*. Typhimurium ST34 clade

We next investigated the extent of mTmV transfer in nature, within *S*. Typhimurium outside of the monophasic *S*. Typhimurium ST34 epidemic clade and diverse isolates of *S. enterica* serotypes. Of 778 *S*. Typhimurium clinical isolates from the UK between 2012 and 2016 [19], the mTmV sequence was present (>90 % coverage with <10 % sequence divergence) in just a single *S*. Typhimurium isolate (strain 107739; Fig. 1, Table S2), predicted to be ST34 but outside the monophasic S. Typhimurium ST34 clade, which may have originated by recombination, consistent with limited transfer outside of the monophasic *S*. Typhimurium ST34 epidemic clade to date.

*S*. Derby and monophasic *S*. Typhimurium ST34 are the two most common serotypes isolated from pigs in the European Union [2]. Therefore, we investigated the presence of *sopE* and mTmV in the whole-genome sequence of 15 *S*. Derby isolated from faecal and environmental samples in Irish pig farms, at which *sopE*-positive monophasic *S*. Typhimurium ST34 were also present [16, 60] (Tables 2 and S1). Four *S*. Derby isolates contained the *sopE* gene and mTmV. Isolates of six additional serotypes (Anatum, Stanley, Infantis, Dublin, London and Tennessee) were also isolated from these pig farms, but none contained the mTmV sequence, while the *sopE* sequence was detected in the genome of two *S *. *enterica* Dublin isolates. However, in *S *. *enterica* Dublin, *sopE* differed by 37 SNPs from the monophasic *S*. Typhimurium ST34 *sopE*, and was present on a lambdoid prophage consistent with previous reports of this serotype [43]. To further test for the distribution of mTmV in *S. enterica *, the nucleotide sequence of mTmV was aligned in the GenBank database (accessed January 2020) using blast. Of 10 811 *S *. *enterica* genome assemblies, 6 contained the mTmV sequence (>90 % sequence coverage with at least 90 % nucleotide identity): 4 *S*. Derby (from Canada, China and France), 1 *S *. *enterica* Brandenburg (from Canada) and 1 *S *. *enterica* California (from China) (Table 3).

**Table 3.** Sequences of non-Typhimurium isolates positive for mTmV prophage in the GenBank database The strain name, serovar, country, source of isolation and accession number are reported together with the nucleotide identity of the prophage over the whole alignment with mTmV sequence compared to strain S04698-09 (accession number NZ_LN999997.1, nucleotides 5 022 794–5 037 238 and 1–24 715), and the nucleotide coordinates of the mTmV prophage in the genome.

Strain	Serovar	Country	Source	Accession no.	Identity (%)	Nucleotide coordinates
SA20113174	Brandenburg	Canada	nk	CP029999.1	99.94	3 532 397–3 571 427
CD-SL01	California	China	Chicken	CP028900.1	99.98	3 610 841–3 649 870
SA20035215	Derby	Canada	nk	CP022494.1	99.97	605 111–644 140
2014LSAL02547	Derby	France	Pork	CP029486.1	99.95	4 041 042–4 080 071
Sa64	Derby	China	nk	CP034250.1	99.91	3 336 809–3 375 855
CFSA231	Derby*	China	Food	CP033350.2	99.98	3 611 673–3 650 702

nk, Source not known.

*Serovar predicted *in silico* using SeqSero-1.2 software [71].

### Lysogenic transfer of mTmV to genotypically diverse *S *. *enterica* serovar isolates

The presence of *sopE* in mTmV in *S*. Derby isolates from pigs and a *S*. Typhimurium isolate from a clinical infection suggested that mTmV was able to transfer to *S. enterica* isolates in nature. Therefore, we investigated the range of mTmV transfer during culture *in vitro*. In order to quantify the lysogenic conversion, we inserted an *aph* or *cat* gene in the intergenic region between the *sopE* 3′ end and the adjacent gene in mTmV in monophasic *S*. Typhimurium ST34 strain 0343A isolated at a pig farm [16].

**Fig. 5.**
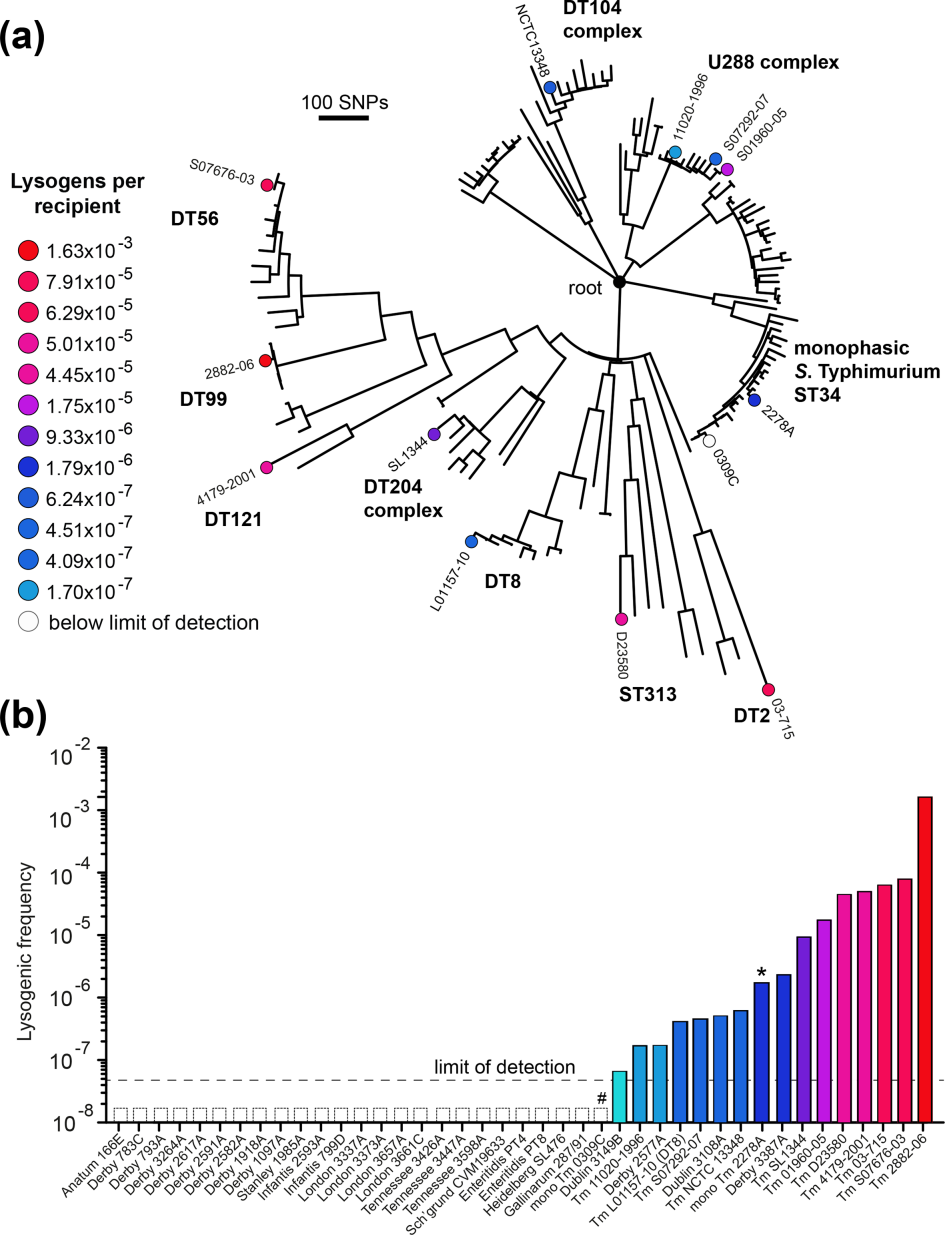
Frequency of mTmV lysogenic conversion in diverse *S*. Typhimurium genotypes and *S *. *enterica* serovars. (a) ML phylogenetic tree of 141 *S*. Typhimurium isolates representative of the genetic diversity within the serovar based on 8402 SNPs with reference to *S*. Typhimurium SL1344. The isolates tested are marked with a filled dot, the colour of the dot refers to the to the frequency of mTmV lysogeny measured as the ratio between the concentration of lysogenic cells and total cells. (b) Bar chart plotting the frequency of lysogenic conversion of mTmV in diverse *S*. Typhimurium genotypes (displayed in a) and diverse *S. enterica* serovars. The dashed line indicates the limit of detection.

Culture of genotypically diverse *S*. Typhimurium isolates with filtered supernatant of monophasic *S*. Typhimurium ST34 strain 0343A mTmV::*kan* or 0343A mTmV::*cat* resulted in the generation of lysogens of the recipient strain. However, the frequency of lysogenic conversion varied by up to 10 000-fold, ranging from 1×10^−7^ to 1×10^−3^ lysogens per recipient (Fig. 5a, b). Lysogeny by mTmV was either undetectable or at low frequency in a collection of 29 isolates representing 11 *S. enterica* serotypes (Table S1). Two *S*. Derby and two *S *. *enterica* Dublin isolates were lysogenized with a frequency ranging between 10^−6^ and 10^−8^ (Fig. 5b).

In each case, the junction between mTmV and the *thrW* locus could be amplified by PCR from genomic DNA of lysogens of *S *. *enterica* Typhimurium, Derby and Dublin, using specific oligonucleotide primers, confirming integration into the *thrW* locus (data not shown). In *S*. Typhimurium strains NCTC 13348 (DT104), D23580 (ST313) and SO7676-03 (DT56), mTmV displaced the TmVII prophage integrated in *thrW* [23], as indicated by the PCR amplification of the mTmV junctions with specific primers.

### HGT and recombination of phage sequences resulted in an increased number of *sopE* gene copies

The *sopE* gene is commonly harboured in single copy by *Salmonella *, although multiple copies in a single genome have been reported [61]. Therefore, we investigated the ratio of short-read sequence that mapped to *sopE* and *sopE2* and identified a cluster of closely related monophasic *S*. Typhimurium ST34 isolates, which had been previously reported [9], that exhibited an increased read mapping to *sopE* (Fig. S1). To further investigate the presence of multiple copies of *sopE* and their genetic context, we generated long-read sequence to the genome of one of these, strain S01569-10. The genome of strain S01569-10 was 4 973 996 bp and included an additional contiguously assembled sequence of 80 kb, absent from strain S04698-09, consistent with an episome (Fig. S1). The isolate had three copies of *sopE* identical to each other and to the *sopE* coding sequence in mTmV, two on the chromosome assembly and a third on the 80 kb episome. The presence of three copies of *sopE* was assessed by PCR (Fig. S2). Two chromosomal copies of *sopE* gene were located in distinct prophages, designated mTmV1569 and Ψm1569. Alignment of the chromosome sequence of S01569-10 and S04698-09 revealed a 2 Mbp inversion with junctions within mTmV and ST64B prophage resulting in two hybrid prophages, one of which, mTmV1569, contained the *sopE* gene (Fig. S3a). Prophage mTmV1569 shared 65 % of the genome with mTmV (99.7 % nucleotide identity), a region that was conserved between mTmV1569, mTmV and mTmV2, indicating a conserved recombination junction for mTmV1569 and mTmV2 (Fig. S3b). A second chromosomal copy of *sopE* was located within a region of 21 kb that is a mosaic of three non-contiguous regions of mTmV1569, and was designated Ψm1569 (pseudo-phage mTmV1569) (Fig. S3b). The Ψm1569 prophage was present at the beginning of the chromosome in the 5′ intergenic region of the *thrA* gene (nucleotide coordinates 27–20 540) (Fig. S3a). Comparative genome analyses showed that mTmV and the related variants mTmV2, mTmV1569 and Ψm1569 are P27-like bacteriophages.

A third copy of the *sopE* gene was present in strain S01569-10 on an 81.1 kb prophage plasmid, designated pS01569. The presence and the circular nature of the genetic element were confirmed by PCR amplification with specific oligonucleotide primers with genomic DNA prepared from strain S01569-10 (data not shown). pS01569 shared 99 % nucleotide sequence identity over 89 % of the sequence with plasmids from four *Shigella dysenteriae* isolates (accession numbers CP026808.1, CP026821.1, CP026816.1 and CP026818.1) (Fig. S3c). pS01569 was predicted to be a functional phage based on sequence homology using phaster software. Over the 70 % of the pS01569 exhibited 90.5 % mean nucleotide sequence identity with the *Escherichia coli* bacteriophage D6, which was also on a phage plasmid (accession number MF356679.1) [62] (Fig. S3c). Genes involved in phage replication and plasmid maintenance were conserved between pS01569 and D6 bacteriophage. pS01569 was rare in monophasic *S*. Typhimurium ST34. In addition to its presence in five phylogenetically related isolates from pigs, poultry and human clinical infections in the UK (Fig. S1), pS01569-related sequences were also detected in 5 of 737 human clinical isolates from the UK of which only 2 were closely related (Fig. 1, Table S2).

## Discussion

The *sopE* gene has a sporadic distribution within the genus *Salmonella *, present in isolates from approximately half of *S. enterica* subspecies I serotypes tested in one study [63]. However, within serotypes where the *sopE* gene was detected, in many cases not all isolates of the serotype contained the gene [54], reflecting its presence on MGEs [64]. In *S*. Typhimurium, *sopE* was present in MDR epidemic strains of the DT204 complex that emerged in Europe in around the year 1974 and the currently dominant monophasic *S*. Typhimurium ST34 [9, 54]. Our analysis of *S*. Typhimurium isolates from human clinical infections in England and Wales spanning the years 2012 to 2016 indicated that *sopE* is rare in this serotype. The *sopE* gene was present in 21 isolates outside of the monophasic *S*. Typhimurium ST34 clade, in 7 of 74 phylogroups of a similar level of root-to-tip sequence divergence to that of the monophasic *S*. Typhimurium ST34 phylogroup. Most of the *sopE*-positive isolates from outside the DT204 complex or monophasic *S*. Typhimurium ST34 clades were isolates on single extended branches on the phylogenetic tree, consistent with their infrequent association with human infection. Despite the rarity of *sopE* in *S*. Typhimurium, this gene has been associated with two of the five dominant MDR strains associated with livestock and human infection since around the year 1965 [4].

Acquisition of AMR genes frequently precedes spread and clonal expansion of bacterial pathogens, for example, the *S*. Typhimurium ST313 lineages in sub-Saharan Africa and the pandemic *S*. Typhimurium DT104 clone [11, 65, 66]. The monophasic *S*. Typhimurium ST34 epidemic clone also acquired genes conferring resistance to multiple antibiotics and the transition metals copper and arsenic [19, 67]. These evolutionary events appear to be critical for the entry of the clone into a niche within which clonal expansion is possible. However, we report HGT that occurred subsequently to clonal expansion which appears to have altered the course of the epidemic. The distribution of *sopE* within the monophasic *S*. Typhimurium ST34 population was consistent with acquisition on multiple occasions, and time-scaled phylogenetic analysis revealed that this occurred around the time that the epidemic was first reported in the UK. The common ancestor of monophasic *S*. Typhimurium ST34 in isolates from UK pigs was around 1995, with the acquisition of *sopE* dating from around 2002 to 2010 in our strain collection. Acquisition of the CTX-M gene by an *E *. *coli* ST131 clone that encoded an extended spectrum β-lactamase may have resulted in clonal expansion of a sub-clone globally, but this was not tested directly [68].

Human clinical isolates of monophasic *S*. Typhimurium ST34 with the *sopE* gene exhibited increased branching in the population structure of UK pig isolates and human clinical isolates. This was quantified by a significantly lower patristic distance of *sopE*-positive human clinical isolates. In a clonally expanding population in which all members of that population have an equal chance of being sampled despite sequence polymorphisms and HGT, we would expect that the patristic distance of *sopE*-positive and -negative isolates would be equal. Lower patristic distance genome sequence suggested that *sopE*-positive isolates were more likely to be present in our sampled population. There are several possible reasons for this, including: (i) increased frequency of *sopE*-positive isolates in pigs due to enhanced fitness, (ii) increased frequency of transmission to the human population, and (iii) increased severity of disease resulting in a greater likelihood that a stool is submitted for testing. The first is possible since a lower patristic distance was also observed for pig isolates, although this did not reach significance due to the relatively low number of genome sequences analysed. Low patristic distance is used as one of the criteria for the identification of outbreaks based on whole-genome sequences [57] and, therefore, it is possible that the decreased patristic distance is due to a greater number of outbreaks due to *sopE*-positive monophasic *S*. Typhimurium ST34. Increased transmission rate between pigs, and between pigs and humans, is consistent with the boost in intestinal colonization of *S*. Typhimurium expressing SopE due to nitrate respiration observed in mice [27], although it is not known if this also occurs in the pig host.

The *sopE* gene was predominantly associated with prophage mTmV in the monophasic *S*. Typhimurium ST34, although recombination of mTmV and other phage, and HGT, resulted in mosaic or entirely novel prophage carrying the gene. Recombination with a lambda-like phage gave rise to mTmV2 that was largely restricted to monophasic *S*. Typhimurium ST34 isolates from Italy [24]. In one cluster of monophasic *S*. Typhimurium ST34 from the UK, an inversion of the chromosome had resulted in generation of mosaic prophage, suggesting that this could be one mechanism for the generation of novel phage carrying the *sopE* gene. In this same cluster of isolates, an identical *sopE* allele was present on a prophage as part of an episome, suggesting that this was likely to have arisen by HGT of the *sopE* gene and flanking sequence. The mechanism of HGT is not known, but may involve a recombinase that is encoded adjacent to the *sopE* gene. The recombinase has similarity to the *hin* gene, which encodes a site-specific recombinase [69]. HGT of the *sopE* gene resulted in an increase in copy number, up to three, in some isolates. An increase in copy number was previously reported in *S *. *enterica* Heidelberg isolates responsible for a large multistate outbreak in the USA in 2011 associated with the consumption of turkey meat [61], although the potential impact on the expression level of SopE and interaction with host cells is not known.

Despite the frequent acquisition of mTmV by monophasic *S*. Typhimurium ST34 during epidemic spread, the prophage was detected in just a single *S*. Typhimurium isolate from outside of this clade. However, lysogenic transfer of mTmV occurred into genotypically diverse strains of *S*. Typhimurium *in vitro* at a variable frequency, in some cases greater than the rate of transfer to monophasic *S*. Typhimurium ST34 lacking the prophage. Lysogenic transfer of mTmV to isolates from other serotypes of *S. enterica* was considerably lower, in most cases below the limit of detection. Nonetheless, mTmV carrying the *sopE* gene was present in six whole-genome sequences in available databases, 0.06 % of genomes, indicating that transfer outside of monophasic *S*. Typhimurium ST34 has occurred in nature. One possibility is that lysogenic transfer occurred during coinfection in an animal reservoir, which may be promoted by inflammation, as reported previously for SopE phage [70]. This possibility was supported by the observation that mTmV was most commonly associated with whole-genome sequence of isolates of *S *. *enterica* Derby which, together with monophasic *S*. Typhimurium ST34, is the most common serotype associated with farmed pigs [2]. Furthermore, in two pig farms from which both *S*. Derby and monophasic *S*. Typhimurium ST34 strains were isolated [16], several isolates of both serotypes contained identical mTmV prophage in their genome. Considering the paucity of mTmV reported outside of the monophasic *S*. Typhimurium ST34 clade, this strongly suggested that they were acquired on the farm.

Whole-genome sequence of bacterial pathogens reveals changes in population structure in response to microevolution during the emergence and clonal expansion of epidemic clones of bacterial pathogens. Sequence polymorphisms associated with clonal expansion of sub-populations pinpoint potential drivers of evolutionary and epidemiological dynamics. We report one example of this that involved HGT of the *sopE* gene on multiple occasions and at a remarkable rate that may have resulted in increased fitness. This work identified a target for subsequent research in epidemiology and bacterial pathogenesis that will directly address the role of SopE in the context of the pig zoonotic reservoir, and transmission to the human population through the food chain.

## Supplementary Data

Supplementary material 1

Supplementary material 2

## References

[1] Majowicz SE, Musto J, Scallan E, Angulo FJ, Kirk M (2010). The global burden of nontyphoidal *Salmonella* gastroenteritis. Clin Infect Dis.

[2] European Food Safety Authority and European Centre for Disease Prevention and Control (EFSA and ECDC) (2019). The European Union one health 2018 zoonoses report. EFSA J.

[3] CDC (2018). National Enteric Disease Surveillance: Salmonella Annual Report, 2016.

[4] Rabsch W, Truepschuch S, Windhorst D, Gerlach RG (2011). Typing Phages and Prophages of Salmonella.

[5] Rabsch W, Tschäpe H, Bäumler AJ (2001). Non-typhoidal salmonellosis: emerging problems. Microbes Infect.

[6] Marin C, D’Auria G, Martínez-Priego L, Marco-Jiménez F (2019). Draft genome sequences of 12 monophasic *Salmonella enterica* subsp. enterica serotype Typhimurium 1,4,[5],12:i:− strains isolated from wild griffon vultures in Eastern Spain. Microbiol Resour Announc.

[7] EFSA Panel on Biological Hazards (BIOHAZ) (2010). Scientific opinion on monitoring and assessment of the public health risk of “*Salmonella* Typhimurium-like” strains. EFSA J.

[8] Rabsch W, Simon S, Humphrey T (2013). Public Health Aspects of *Salmonella* Infections. In: *Salmonella in Domestic Animals*.

[9] Petrovska L, Mather AE, AbuOun M, Branchu P, Harris SR (2016). Microevolution of monophasic *Salmonella* Typhimurium during epidemic, United Kingdom, 2005–2010. Emerg Infect Dis.

[10] Leekitcharoenphon P, Hendriksen RS, Le Hello S, Weill F-X, Baggesen DL (2016). Global genomic epidemiology of *Salmonella enterica* serovar Typhimurium DT104. Appl Environ Microbiol.

[11] Mather AE, Reid SWJ, Maskell DJ, Parkhill J, Fookes MC (2013). Distinguishable epidemics of multidrug-resistant *Salmonella* Typhimurium DT104 in different hosts. Science.

[12] Hauser E, Tietze E, Helmuth R, Junker E, Blank K (2010). Pork contaminated with *Salmonella enterica* serovar 4,[5],12:i:-, an emerging health risk for humans. Appl Environ Microbiol.

[13] García P, Malorny B, Rodicio MR, Stephan R, Hächler H (2016). Horizontal acquisition of a multidrug-resistance module (R-type ASSuT) is responsible for the monophasic phenotype in a widespread clone of *Salmonella* serovar 4,[5],12:i:-. Front Microbiol.

[14] Mather AE, Phuong TLT, Gao Y, Clare S, Mukhopadhyay S (2018). New variant of multidrug-resistant *Salmonella enterica* serovar Typhimurium associated with invasive disease in immunocompromised patients in Vietnam. mBio.

[15] Biswas S, Li Y, Elbediwi M, Yue M (2019). Emergence and dissemination of *mcr*-carrying clinically relevant *Salmonella* Typhimurium monophasic clone ST34. Microorganisms.

[16] Tassinari E, Duffy G, Bawn M, Burgess CM, McCabe EM (2019). Microevolution of antimicrobial resistance and biofilm formation of *Salmonella* Typhimurium during persistence on pig farms. Sci Rep.

[17] Sun H, Wan Y, Du P, Bai L (2020). The epidemiology of monophasic *Salmonella* Typhimurium. Foodborne Pathog Dis.

[18] Polz MF, Alm EJ, Hanage WP (2013). Horizontal gene transfer and the evolution of bacterial and archaeal population structure. Trends Genet.

[19] Branchu P, Charity OJ, Bawn M, Thilliez G, Dallman TJ (2019). SGI-4 in monophasic *Salmonella* Typhimurium ST34 is a novel ICE that enhances resistance to copper. Front Microbiol.

[20] Mourão J, Novais C, Machado J, Peixe L, Antunes P (2015). Metal tolerance in emerging clinically relevant multidrug-resistant *Salmonella enterica* serotype 4,[5],12:i:- clones circulating in Europe. Int J Antimicrob Agents.

[21] Dębski B (2016). Supplementation of pigs diet with zinc and copper as alternative to conventional antimicrobials. Pol J Vet Sci.

[22] Harrison E, Brockhurst MA (2017). Ecological and evolutionary benefits of temperate phage: what does or doesn't kill you makes you stronger. Bioessays.

[23] Bawn M, Thilliez G, Wheeler N, Kirkwood M, Petrovska L (2019). Evolution of *Salmonella enterica* serotype Typhimurium driven by anthropogenic selection and niche adaptation. bioRxiv.

[24] Palma F, Manfreda G, Silva M, Parisi A, Barker DOR (2018). Genome-wide identification of geographical segregated genetic markers in *Salmonella enterica* serovar Typhimurium variant 4,[5],12:i:-. Sci Rep.

[25] Hardt WD, Chen LM, Schuebel KE, Bustelo XR, Galán JE (1998). *S. typhimurium* encodes an activator of Rho GTPases that induces membrane ruffling and nuclear responses in host cells. Cell.

[26] Keestra AM, Winter MG, Auburger JJ, Frässle SP, Xavier MN (2013). Manipulation of small Rho GTPases is a pathogen-induced process detected by NOD1. Nature.

[27] Lopez CA, Winter SE, Rivera-Chávez F, Xavier MN, Poon V (2012). Phage-mediated acquisition of a type III secreted effector protein boosts growth of *Salmonella* by nitrate respiration. mBio.

[28] Vonaesch P, Sellin ME, Cardini S, Singh V, Barthel M (2014). The *Salmonella *Typhimurium effector protein SopE transiently localizes to the early SCV and contributes to intracellular replication. Cell Microbiol.

[29] Datsenko KA, Wanner BL (2000). One-step inactivation of chromosomal genes in *Escherichia coli* K-12 using PCR products. Proc Natl Acad Sci USA.

[30] Chan W, Costantino N, Li R, Lee SC, Su Q (2007). A recombineering based approach for high-throughput conditional knockout targeting vector construction. Nucleic Acids Res.

[31] Kirkwood M, Vohra P, Bawn M, Thilliez G, Pye H (2020). Ecological niche adaptation of a bacterial pathogen associated with reduced zoonotic potential. bioRxiv.

[32] Kröger C, Dillon SC, Cameron ADS, Papenfort K, Sivasankaran SK (2012). The transcriptional landscape and small RNAs of *Salmonella enterica* serovar Typhimurium. Proc Natl Acad Sci USA.

[33] McClelland M, Sanderson KE, Spieth J, Clifton SW, Latreille P (2001). Complete genome sequence of *Salmonella enterica* serovar Typhimurium LT2. Nature.

[34] Seemann T (2015). https://github.com/tseemann/snippy.

[35] Li H, Durbin R (2009). Fast and accurate short read alignment with Burrows-Wheeler transform. Bioinformatics.

[36] Garrison E, Marth G (2012). Haplotype-based variant detection from short-read sequencing. arXiv.

[37] Danecek P, Auton A, Abecasis G, Albers CA, Banks E (2011). The variant call format and VCFtools. Bioinformatics.

[38] Croucher NJ, Page AJ, Connor TR, Delaney AJ, Keane JA (2015). Rapid phylogenetic analysis of large samples of recombinant bacterial whole genome sequences using Gubbins. Nucleic Acids Res.

[39] Stamatakis A (2014). RAxML version 8: a tool for phylogenetic analysis and post-analysis of large phylogenies. Bioinformatics.

[40] Paradis E, Claude J, Strimmer K (2004). APE: analyses of phylogenetics and evolution in R language. Bioinformatics.

[41] Wailan AM, Coll F, Heinz E, Tonkin-Hill G, Corander J (2019). rPinecone: define sub-lineages of a clonal expansion via a phylogenetic tree. Microb Genom.

[42] Kozlov AM, Darriba D, Flouri T, Morel B, Stamatakis A (2019). RAxML-NG: a fast, scalable and user-friendly tool for maximum likelihood phylogenetic inference. Bioinformatics.

[43] Mirold S, Rabsch W, Tschäpe H, Hardt WD (2001). Transfer of the *Salmonella* type III effector sopE between unrelated phage families. J Mol Biol.

[44] Yu G, Smith DK, Zhu H, Guan Y, Lam TT‐Y (2017). GGTREE: an R package for visualization and annotation of phylogenetic trees with their covariates and other associated data. Methods Ecol Evol.

[45] Letunic I, Bork P (2019). Interactive tree of life (iTOL) v4: recent updates and new developments. Nucleic Acids Res.

[46] Rambaut A (2015). http://tree.bio.ed.ac.uk/software/figtree/.

[47] Hunt M, Mather AE, Sánchez-Busó L, Page AJ, Parkhill J (2017). ARIBA: rapid antimicrobial resistance genotyping directly from sequencing reads. Microb Genom.

[48] Inouye M, Dashnow H, Raven L-A, Schultz MB, Pope BJ (2014). SRST2: rapid genomic surveillance for public health and hospital microbiology Labs. Genome Med.

[49] Altschul SF, Madden TL, Schäffer AA, Zhang J, Zhang Z (1997). Gapped BLAST and PSI-BLAST: a new generation of protein database search programs. Nucleic Acids Res.

[50] Guy L, Kultima JR, Andersson SGE (2010). genoPlotR: comparative gene and genome visualization in R. Bioinformatics.

[51] Didelot X, Croucher NJ, Bentley SD, Harris SR, Wilson DJ (2018). Bayesian inference of ancestral dates on bacterial phylogenetic trees. Nucleic Acids Res.

[52] Revell LJ (2012). phytools: an R package for phylogenetic comparative biology (and other things). Methods Ecol Evol.

[53] R Core Team (2019). R: a language and environment for statistical computing.

[54] Hopkins KL, Threlfall EJ (2004). Frequency and polymorphism of sopE in isolates of *Salmonella enterica* belonging to the ten most prevalent serotypes in England and Wales. J Med Microbiol.

[55] Buu TN, van Soolingen D, Huyen MNT, Lan NTN, Quy HT (2012). Increased transmission of *Mycobacterium tuberculosis* Beijing genotype strains associated with resistance to streptomycin: a population-based study. PLoS One.

[56] Wertheim JO, Oster AM, Johnson JA, Switzer WM, Saduvala N (2017). Transmission fitness of drug-resistant HIV revealed in a surveillance system transmission network. Virus Evol.

[57] Coipan CE, Dallman TJ, Brown D, Hartman H, van der Voort M (2020). Concordance of SNP- and allele-based typing workflows in the context of a large-scale international *Salmonella* enteritidis outbreak investigation. Microb Genom.

[58] Dallman T, Ashton P, Schafer U, Jironkin A, Painset A (2018). SnapperDB: a database solution for routine sequencing analysis of bacterial isolates. Bioinformatics.

[59] Boyd EF, Wang FS, Beltran P, Plock SA, Nelson K (1993). *Salmonella* reference collection B (SARB): strains of 37 serovars of subspecies I. J Gen Microbiol.

[60] Burns AM (2015). *Assessing and managing the risk posed by Salmonella in pig feed.* PhD Thesis, Waterford Institute of Technology, Ireland.

[61] Hoffmann M, Zhao S, Pettengill J, Luo Y, Monday SR (2014). Comparative genomic analysis and virulence differences in closely related *Salmonella enterica* serotype Heidelberg isolates from humans, retail meats, and animals. Genome Biol Evol.

[62] Gilcrease EB, Casjens SR (2018). The genome sequence of *Escherichia coli* tailed phage D6 and the diversity of *Enterobacteriales* circular plasmid prophages. Virology.

[63] Prager R, Mirold S, Tietze E, Strutz U, Knüppel B (2000). Prevalence and polymorphism of genes encoding translocated effector proteins among clinical isolates of *Salmonella enterica*. Int J Med Microbiol.

[64] Mirold S, Rabsch W, Rohde M, Stender S, Tschäpe H (1999). Isolation of a temperate bacteriophage encoding the type III effector protein SopE from an epidemic *Salmonella typhimurium* strain. Proc Natl Acad Sci USA.

[65] Okoro CK, Kingsley RA, Connor TR, Harris SR, Parry CM (2012). Intracontinental spread of human invasive *Salmonella* Typhimurium pathovariants in sub-Saharan Africa. Nat Genet.

[66] Kingsley RA, Msefula CL, Thomson NR, Kariuki S, Holt KE (2009). Epidemic multiple drug resistant *Salmonella* Typhimurium causing invasive disease in sub-Saharan Africa have a distinct genotype. Genome Res.

[67] Lucarelli C, Dionisi AM, Filetici E, Owczarek S, Luzzi I (2012). Nucleotide sequence of the chromosomal region conferring multidrug resistance (R-type ASSuT) in *Salmonella* Typhimurium and monophasic *Salmonella* Typhimurium strains. J Antimicrob Chemother.

[68] Price LB, Johnson JR, Aziz M, Clabots C, Johnston B (2013). The epidemic of extended-spectrum-β-lactamase-producing *Escherichia coli* ST131 is driven by a single highly pathogenic subclone, H30-Rx. mBio.

[69] Johnson RC (2015). Site-Specific DNA inversion by serine recombinases. Microbiol Spectr.

[70] Diard M, Bakkeren E, Cornuault JK, Moor K, Hausmann A (2017). Inflammation boosts bacteriophage transfer between *Salmonella* spp. Science.

[71] Zhang S, Yin Y, Jones MB, Zhang Z, Deatherage Kaiser BL (2015). *Salmonella* serotype determination utilizing high-throughput genome sequencing data. J Clin Microbiol.

