# Supplementary material for: Whole-genome epidemiology links phage-mediated acquisition of a virulence gene to the clonal expansion of a pandemic Salmonella enterica serovar Typhimurium clone
Source: Microb Genom. 2020 Oct 28;6(11):mgen000456. doi: 10.1099/mgen.0.000456 (PMC7725340; doi:10.1099/mgen.0.000456)
Supplement: Supplementary material 2 [file mgen-6-456-s001.pdf]

Whole-genome epidemiology links phage-mediated acquisition of a virulence gene to the clonal expansion of a pandemic *Salmonella* Typhimurium clone

Eleonora Tassinari, Matt Bawn, Gaetan Thilliez, Oliver Charity, Luke Acton, Mark Kirkwood, Liljana Petrovska, Timothy Dallman, Catherine M. Burgess, Neil Hall, Geraldine Duffy and Robert A. Kingsley

Supplementary Figures

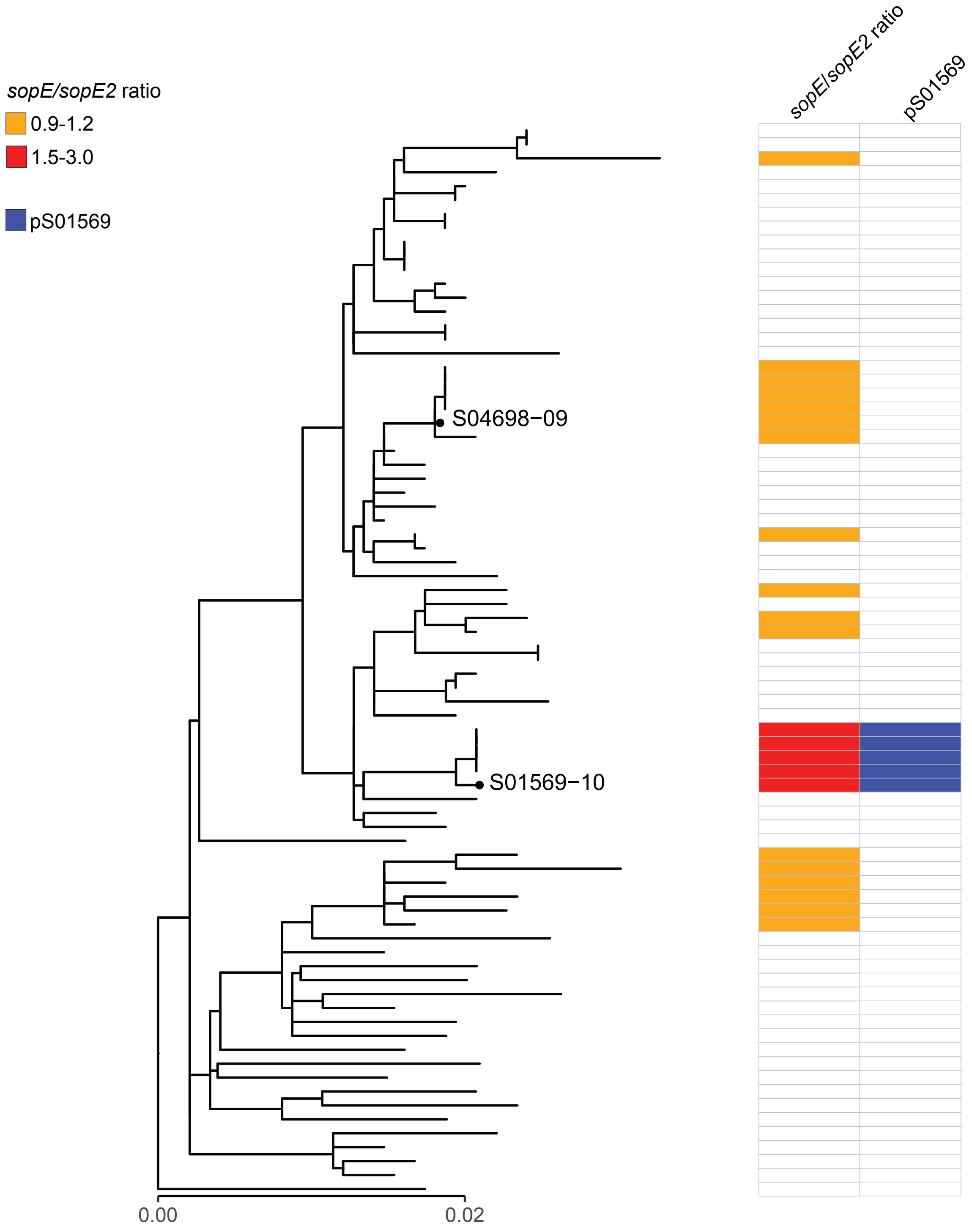

Supplementary Figure 1. Variation of sopE/sopE2 sequence read ratio and distribution of pS01569 prophage plasmidin monophasic S. Typhimurium ST34. The maximum-likelihood tree was based on 1,516 SNPs identified in monophasic S. Typhimurium ST34 previously described (9) with reference to S. Typhimurium SL1344. The location of strains S04698-09 and S01569-10 is indicated. sopE was detected in 22 isolates with SRST2 (48). The ratio of the number of reads mapping to sopE an sopE2 is reported. The distribution of the pS01569 prophage plasmid across the phylogenetic tree is shown.

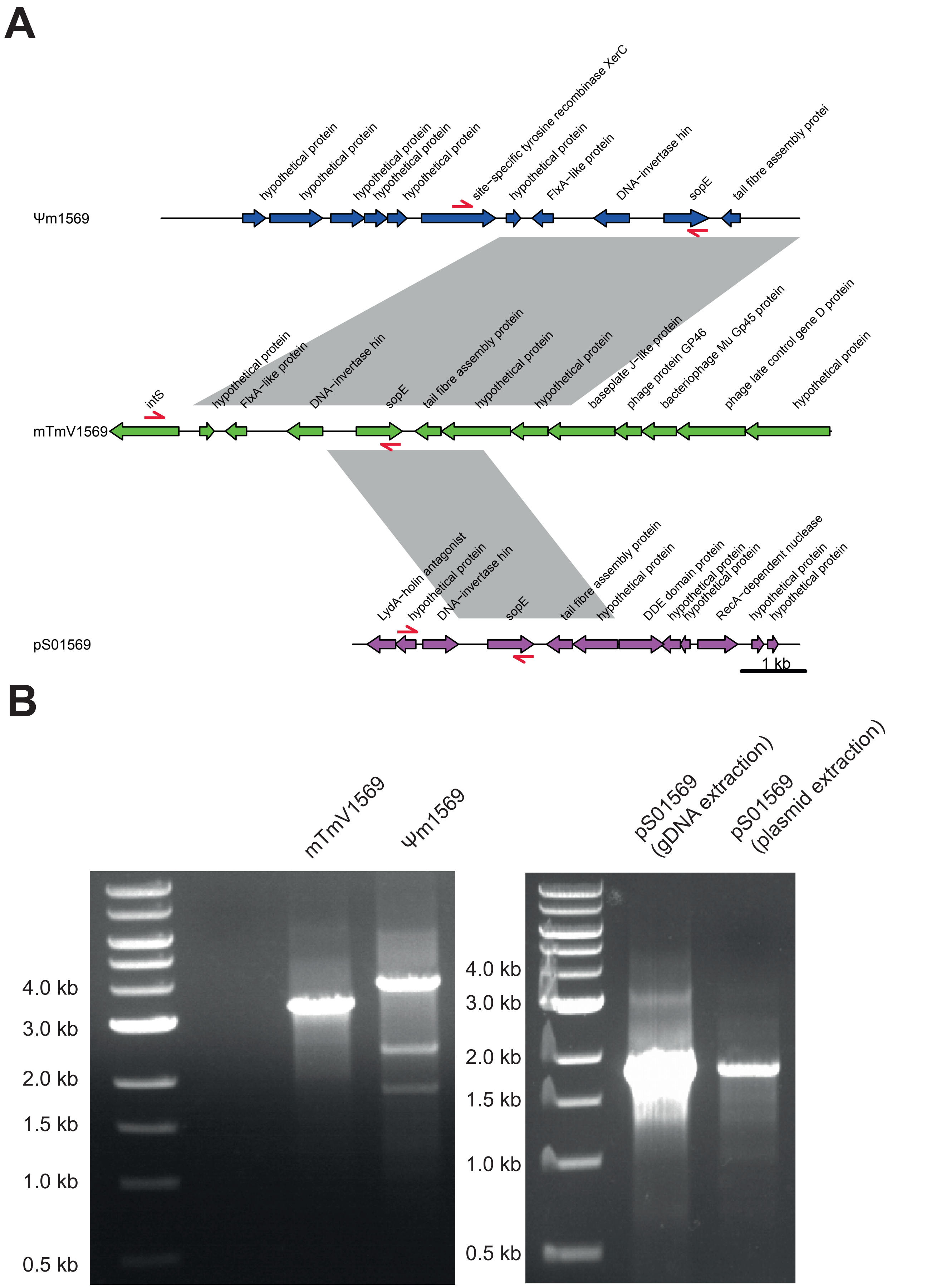

Supplementary Figure 2. PCR amplification of the three copies of sopE in monophasic S. Typhimurium S01569-10. A) Multiple sequence alignment of the sopE genes and flanking regions in Ψm1569, mTmV1569 and pS01569 in monophasic S. Typhimurium S01569-10 showing the conserved sequences and the annealing sites of the oligonucleotides (red half arrows) used for the PCR reaction. B) Agarose gel showing the amplicons obtained. The expected amplicon size associated with the amplification of sopE in mTmV1569, Ψm1569 and pS01569 was 3.2 kb, 3.9 kb and 1.6 kb, respectively.

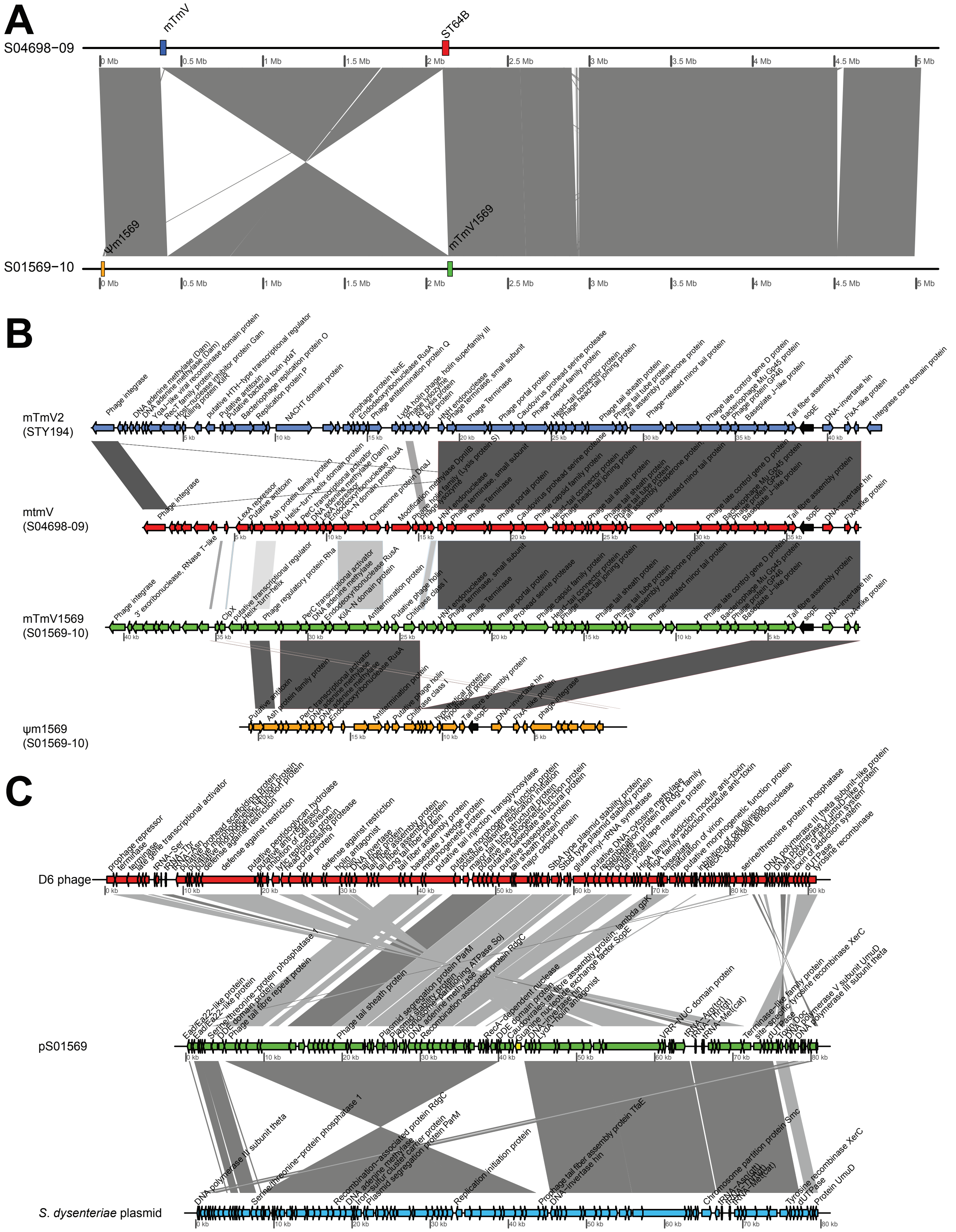

Supplementary Figure 3. Comparative genomic analyses of bacteriophages encoding sopE in monophasic S. Typhimurium ST34. A) Whole-genome comparison (S04698-09 and S01569-10) showing a 2 Mb DNA inversion in S01569-10 following recombination between mTmV and ST64B prophages. mTmV (blue box) and ST34B (red box) prophages in strain S04698-09, the recombinant prophage mTmV1569 (green box) in strain S01569-10, and the location of the Ψm1569 prophage sequence (orange box) in strain S01569-10 are shown. Nucleotide identity: ≥90% (grey shade). B) Genome comparison between mTmV2 (strain STY194), mTmV (strain S04698-09) and the sopE phages integrated in the chromosome of strain S01569-10. The coding sequences for each phage genome are displayed as filled arrow and are annotated with the corresponding gene product; the genes which are not annotated encode for hypothetical proteins. sopE gene (black arrow). Shades of grey representnucleotide identity: dark grey: 95-100% identity, mid-grey: 85-94% identity; light-grey: 80-93% identity. C) Alignment between E. coli phage D6, pS01569 (strain S01569-10) and S. dysenteriae plasmid (strain 204/96). The coding sequences for each phage genome are displayed as filled arrow and are annotated with the corresponding gene product; when no text is present the gene encodes for hypothetical protein. Dark grey: 90-100% nucleotide identity; light-grey: 73-89% nucleotide identity.
